# Achieving recommended endotracheal tube intracuff pressure: a randomized control study comparing loss of resistance syringe to pilot balloon palpation (the LOR-ICP trial, nct02294422)

**DOI:** 10.1186/2197-425X-3-S1-A931

**Published:** 2015-10-01

**Authors:** F Bulamba, A Kwizera, L Ssemogerere, N Ayupo, C Kojjo, A Kintu, LOR-ICP TRIAL Group

**Affiliations:** Makerere University, Anaesthesia, Kampala, Uganda; International Hospital Kampala, Intensive Care, Kampala, Uganda

## Introduction

It has been agreed that the safe endotracheal tube intracuff pressures ranges from 20cmH2O to 30cmH2O with pressures above that range being dangerous to the patient even for a short time. While there are many different methods of inflation, the cuff manometer is considered the standard. However, its applicability in clinical practice is marred with numerous hindrances. Without the cuff manometer, anaesthesia providers employ a varied range of subjective methods to inflate and estimate optimal ETT cuff pressure. The most common method used is the palpation of the pilot balloon. This method has been shown to administer high ETT cuff pressures which are associated with both early and late post extubation airway symptoms.

## Objectives

The study evaluated the loss of resistance (LOR) method through a single blinded, randomized control trial with an objective of determining the efficacy and safety of the LOR method at estimating ETT intracuff pressures against the pilot balloon palpation (PBP) method in elective surgical adults intubated with high volume low pressure cuff ETT.

## Methods

With approval from the institution IRB, we randomized 178 patients scheduled to undergo general anesthesia at a teaching hospital into two equal groups. These patients had their ETT intracuff pressures estimated by the LOR method or the PBP method and measurements taken with a cuff manometer. Patients were later reviewed for post extubation airway complaints.

## Results

Of the pressures in the recommended range, only 25.3% were by the PBP method vs 74.7% by the LOR with a statistically significant positive mean difference (MD) 0.47191, pvalue = 0.000 (95% CI 0.343-0.602). Overall, the incidence of post extubation airway complaints was 74.7% in this study population. The PBP group had an incidence of 73.0% compared to 55.0% of the LOR group. The incidence of airways complaints was only significantly increased by the level of anesthesia provider AOR = **0.4** (pvalue =**0.018**, 95% CI **0.2-0.8**).

## Conclusions

The LOR method was superior to the PBP at administering pressures in the recommended range. The incidence of post extubation airway complaints in the study population was high. The only associated factor was level of provider.

The LOR method does not replace the manometer as the standard but provides an option that is fairly devoid of high cuff pressures especially for a setting with no effective use of the manometer. This method should be evaluated in the Intensive Care Unit and Emergency department.

## Grant Acknowledgment

We had no grant or any financial support towards the conduct of this study.
